# Prevalence of *Toxoplasma gondii* Infection among Healthy Blood Donors in Northeast of Iran

**Published:** 2017

**Authors:** Saeed SADOOGHIAN, Hossein MAHMOUDVAND, Mohammad Ali MOHAMMADI, Naser NAZARI SARCHESHMEH, Amir TAVAKOLI KARESHK, Hossein KAMIABI, Naser ZIA-ALI

**Affiliations:** 1. Dept. of Medical Parasitology and Mycology, Kerman University of Medical Sciences, Kerman, Iran; 2. Dept. of Medical Parasitology and Mycology, Lorestan University of Medical Sciences, Khorramabad, Iran; 3. Blood Transfusion Organization of Khorasan Razavi, Mashhad, Iran

**Keywords:** Toxoplasmosis, Blood transfusion, IgG antibody, IgM antibody, Nested-PCR, Iran

## Abstract

**Background:**

This cross-sectional investigation aimed to evaluate the prevalence of IgM and IgG anti-*Toxoplasma gondii* antibodies and the associated risk factors among healthy blood donors in Khorasan Razavi Province, northeast of Iran from Nov 2014 to May 2015.

**Methods:**

Overall, 491 serum samples from apparently healthy blood donors referred the six biggest blood centers in Razavi Khorasan, Iran, were screened for IgG and IgM anti-*T. gondii* antibodies by enzyme-linked immunosorbent assay (ELISA). A structured questionnaire was used to obtain information on risk factors for *T. gondii* infection. Nested PCR was also used to detect DNA of *T. gondii* in the IgM-positive samples by using of B1 and RE (Repetitive Element) as marker for amplifying fragment size of 531 bp and 164 bp in PCR method.

**Results:**

Totally, 200 (40.7%) samples were seropositive for anti-*T. gondii* antibodies; 184 (37.5%) donors tested seropositive for only IgG antibody, 8 (1.6%) tested seropositive for both IgM and IgG and 8 (1.6%) were positive for IgM antibody alone. Several risk factors significantly related to *T. gondii* seropositivity in the univariate analysis at *P*<0.05 included age (*P*<0.001), and raw/half-cocked meat consumption (*P*=0.015). *T. gondii* DNA was found in all sixteen IgM-positive samples.

**Conclusion:**

*T. gondii* infection was present among healthy blood donors in northeast of Iran. Thus, it is suggested to design screening programs for preventing transfusion-transmitted toxoplasmosis.

## Introduction

*Toxoplasma gondii*, an obligate intra-cellular parasite, is a generally successful microorganism that infects around 30% of the human population globally (1). The transmission of infection occurs by ingestion of water, vegetables and/or soil contaminated with oocysts from cat feces; or raw/undercooked meat containing tissue cysts, and congenitally (2, 3). *Toxoplasma* infection may be transmitted via the whole blood or white blood cell transfusions or organ transplantation to vulnerable recipients (3, 4).

Among immunocompetent people, toxoplasmosis is generally asymptomatic, whereas, the infection in the immunocompromised individuals such as patients with acquired immunodeficiency syndrome, and neonates can be fatal for their living (5–8). Considering a number of factors for example community and cultural behavior, climate, and form of transmission, the prevalence of toxoplasmosis is varying from 10% to 80% (9–12). In Iran, the prevalence of this infection varies depending on geographical regions was approximately 18% to 70% (13). At present time, there are numerous serological investigations on the prevalence of *T. gondii* antibodies between blood donors in various regions of the world (14–24); however, there are a small number of studies on seroprevalence of toxoplasmosis in healthy blood donors of Iran (25–30).

The present cross-sectional investigation aimed to determine the prevalence of IgM and IgG anti-*T*. *gondii* antibodies and the associated risk factors among healthy blood donors in Razavi Khorasan Province, northeastern Iran. Moreover, as a second objective, to confirm the presence of *T. gondii* DNA and parasitemia in blood donors, all IgM-positive analyzed using molecular tests with diagnostic markers.

## Materials and Methods

### Study design

This cross-sectional study was carried out in the six biggest blood centers of Razavi Khorasan Blood Transfusion Organization (RKBTO) in Razavi Khorasan, Iran. This province covers an area of 144681 km^2^ with the population of nearly 6000000 and is located in the northeast of Iran.

### Sample collection and participants

A total of, 491 serum samples were collected from apparently healthy blood donors referred the six biggest blood centers of RKBTO in Razavi Khorasan, Iran, during the period from Nov 2014 to May 2015.

### Questionnaire

The applied questionnaire anchored in demographic data including age, gender, education, residence, and blood group was prepared before collection of blood samples. Moreover, possible risk factors, such as animal contacts (cats), raw/half-cooked meat consumption (lamb and beef), consumption of raw vegetables and raw egg/milk, gardening or agriculture activity, and blood transfusion were evaluated.

### ELISA test

To evaluate the anti-*T. gondii* antibodies, all the serum samples were tested using the commercially available ELISA kit (Dia.Pro, Italy). Analyses were performed following the manufacturer’s instructions. Based on the ELISA kit, positive results for IgG and IgM were defined as values of ≥500 international units (IU)/ml and index values of ≥0.6, respectively. Range of equivocal results was from 250 to 500 IU/ml and index values of 0.5 to 0.6 were assumed for IgG and IgM, respectively. In addition, negative results were defined as < 250 IU/ml and index values of < 0.5 were considered for IgG and IgM, respectively.

### Molecular study using Nested PCR

Nested PCR was used to detect DNA of *T. gondii* in the IgM-positive samples. DNA was extracted from the buffy coat of all of the IgM positive samples according to the method described elsewhere (27). DNA of each sample was extracted using the DNeasy blood and tissue kit (Bioneer, South Korea) according to manufacturer’s instructions. DNA was stored at −20 °C until further use in PCR analysis. Specific primers related to both regions of B1 and RE (Repetitive Element) were used. PCR primers used for B1 gene amplification are as follows: Pml/S1, 5-TGTTCTGTCCTATCGCAACG (positions 128 to 147); Pml/S2, 5-TCTTCCCAGACGTGGATTTC (positions 152 to 171); Pml/AS1, 5-ACGGATGCAGTTCCTTTCTG (positions 707 to 688); and Pml/AS2, 5-CTCGACAATACGCTGCTTGA (positions 682 to 663). PCR primers used for RE gene amplification are as follows: RE nested PCR1, 5′-TGACTCGGGCCCAGCTGCGT (positions 71 to 90); RE nested PCR1, 5′-CTCCTCCCTTCGTCCAAGCCTCC (positions 490 to 468); RE nested PCR2, 5′-AGGGACAGAAGTCGAAGGGG (positions 187 to 206); and RE nested PCR2, 5′-GCAGCCAAGCCGGAAACATC (positions 350 to 331) (31–33).

Each reaction was carried out in a final volume of 25 μl, containing 1μl of each primer, 10-μl water, 8-μl of master (Ampliqon™) and 5μl of DNA for both B1 and RE gene.

The target B1 gene amplified using the following conditions: one cycle of 3 min initial denaturation at 94 °C followed by 35 cycles of 94 °C for 30 sec, 60 °C for 1 min,72 °C for 2 min and was ended by one cycle of final extension at 30 °C for 1 min. Reaction for RE were started with an initial denaturation at 94 C for 3 min, and then cycled 30 times with denaturation at 94 °C for 30 sec, follow by annealing at 60 °C for 30 sec, and finally an extension step at 72 °C for 2 min follow by 1 min final extension at 30 sec. PCR products of second round of the PCR were loaded onto a 1.5% agarose gel and the results were compared with standard band markers of *T. gondii*, 531 bp.

### Ethics

Ethics Committee of Kerman University of Medical Sciences approved this study. In addition, a written informed consent was obtained from all the participants before blood sampling.

### Statistical analyses

Statistical analysis was carried out using SPSS 17.0 software (Inc., Chicago, IL, USA). Logistic regression models were used to evaluate univariate between *T. gondii* seropositivity and the potential risk factors. Multivariate logistic analysis was performed with the full model, including all potential risk factors in the analyses. In this survey, *P*<0.05 was considered to be statistically significant.

## Results

### Participants

Overall, 491 blood donors were included in the present study; the mean age of the participants was 36.29±4.16 yr old (ranging from 18 to 57 yr old). Most participants were male (93.9%), aged 30–40 yr old, living in urban areas, which had college education or above.

### Seroprevalence of anti-T. gondii antibodies

From 491 blood donors, 200 (40.7%) samples were seropositive for anti-*T. gondii* antibodies; 184 (37.5%) donors tested seropositive for only IgG antibody, 8 (1.6%) tested sero-positive for both IgM and IgG and 8 (1.6%) were positive for IgM antibody alone; indicating the seroprevalence of IgG and IgM anti-*T. gondii* antibodies were 37.5% and 3.2%, respectively. Regarding geographical region, seroprevalence of anti-*T. gondii* IgG antibody in six blood centers including Mashhad, Quchan, Torbat-e Heydarieh, Nishapur, Sabzevar, and Gonabad were 33.5, 35, 50, 41.6, 37.7, and 35% respectively. Seroprevalence of anti-*T. gondii* IgG antibodies were significantly higher among female donors; whereas, no significant difference was observed in the prevalence of IgM anti-*T. gondii* among the female and male donors (*P*=0.811). Statistical analysis also demonstrated that a significant difference in the prevalence of IgG (*P*≤0.001) and IgM (*P*=0.95) anti-*T. gondii* antibodies among different age groups (Table 1).

**Table 1:** Demographic characteristics and *T. gondii* sero-prevalence among healthy blood donors in Razavi Khorasan Province, Iran

***Variables***	***No. (%)***	***IgG positive***	***IgM positive***
**Gender**			
Male	461 (93.9)	(37.3)	(2.4)
Female	30 (6.1)	(40)	16.7
**Age group(yr)**			
18–30	161 (32.8)	36	0
30–40	170 (34.6)	39.4	0.58
40–50	116 (23.6)	34.5	4.3
50–60	42(8.6)	42.8	23.8
>60yrs	2 (4)	0.5	0
**Residential place**			
Urban	421 (85.5)	38.2	2.9
Rural	70 (14.3)	32.9	5.7
**Education**			
Less than diploma	200 (40.7)	29.5	4.5
Diploma and above	291 (59.3)	43	2.4
**Blood type**			
A	150 (30.5)	35.3	4
B	150 (30.5)	33.3	3.3
AB	46 (9.4)	39.1	2.2
O	145 (29.5)	43.4	2.8
**Being in contact with cat**			
No	478 (97.4)	37.7	3.1
Yes	13 (2.6)	308	7.7
**Raw/half-cooked meat consumption**			
No	443 (90.2)	37.7	2.9
Yes	48 (9.8)	35.4	6.3
**Gardening or agriculture**			
No	346 (70.5)	38.2	3.5
Yes	145 (29.5)	35.9	2.8
**Blood transfusion**			
No	478 (97.4)	37.4	3.3
Yes	13 (2.6)	38.5	0

### Risk factors for being anti-T gondii antibodies

Several risk factors significantly related to *T. gondii* seropositivity in the univariate analysis at *P*<0.05 included age (*P*<0.001), and raw/half-cocked meat consumption (*P*<0.01). However, other demographic and risk factors of the blood donors did not show any correlation with *T. gondii* seropositivity (Table 2). The correlation between risk factors and status of anti-*T. gondii* IgG antibodies in the univariate analysis (crude OR) are shown in Table 2.

**Table 2:** Univariate (crude OR) logistic regression analysis of the potential factors associated with *T. gondii* IgG seroprevalence among healthy blood donors in Razavi Khorasan Province, Iran

**Variables**	***IgG Positive***	
	**Crude OR (95% CI)**	***P* value**
**Gender**		
Male	1	-
Female	1.5 (0.7, 3.1)	0.29
**Age groups (yr)**		
18–30	-	-
30–40	-	-
40–50	-	-
50–60	-	-
>60	1.6	0.001*
**Residential place**		
Urban	1	0.64
Rural	1.13 (0.675, 1.9)	
**Education**		
Less than diploma	1	-
Diploma and above	1.5 (1.15, 2.3)	0.14
**Blood type**		
A	1	-
B	0.97 (0.16, 1.5)	0.91
AB	0.59 (0.29, 1.21)	0.15
O	0.84 (0.52, 1.34)	0.46
**Being in contact with cat**		
No	1	-
Yes	1.44 (0.47, 4.36)	0.51
**Raw/half-cooked meat consump-**		
**tion**	1	-
No	2.1 (1.14, 3.88)	0.018*
Yes		
**Gardening or agriculture**		
No	1	-
Yes	1.16 (0.78, 1.73)	0.45
**Eating uncooked vegetables** No		
No	1	-
Yes	1.27 (0.65, 2.46)	0.49
**Blood transfusion**		
No	1	-
Yes	0.49 (0.13,1.81)	0.29

In multiple logistic regression, age (*P*<0.001), and raw/half-cocked meat consumption were independent risk factors for *Toxoplasma* seropositivity.

### Nested-PCR

**Fig. 1:**
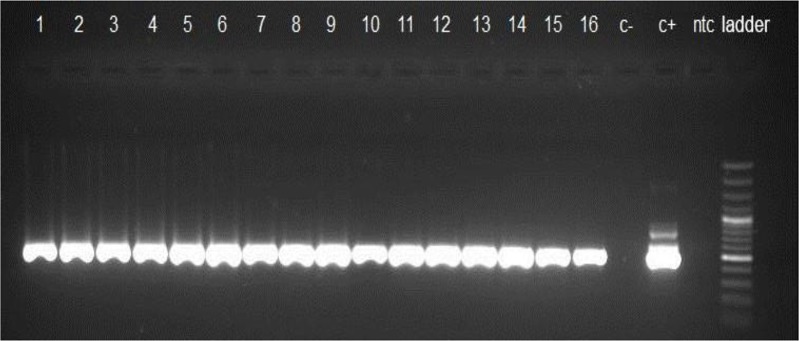
B1-nested PCR analysis of *T. gondii* DNA (531 bp). Lane 1–16:IgM-positive blood samples; C-: negative control (D.W); C+: positive control (*T.gondii* RH strain); ntc: negative test control (*T.gondii* negative sample); (ladder (100 bp)

**Fig. 2:**
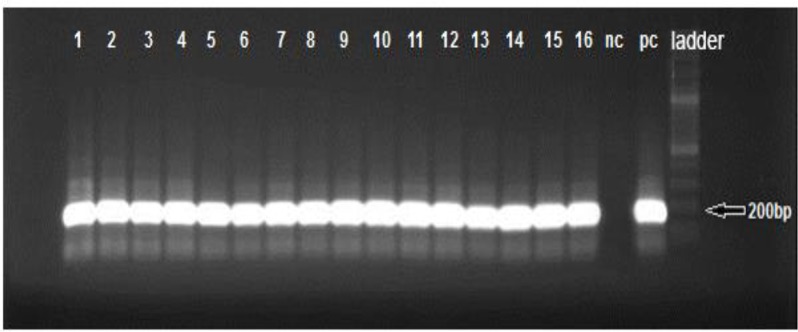
RE-nested PCR analysis of *T. gondii* DNA (164bp). Lane 1–16:IgM-positive blood samples; nc: negative control (D.W); pc: positive control (*T.gondii* RH strain); ladder (100 bp)

To confirm the presence of *T. gondii* DNA in IgM-positive blood donors, all IgM-positive were analyzed using B1 and RE primers (Fig. 1 and 2). The findings revealed that *T. gondii* DNA was found in all sixteen IgM-positive samples. For all of samples, fragment of about 531, 164 bp were successfully PCR-amplified within B1 and RE genes, respectively. Therefore, there was a statistically significant association between IgM positivity and PCR results (*P*<0.05).

## Discussion

Toxoplasmosis in immunocompromised persons could cause severe manifestations such as central nervous system involvements as encephalitis and some brain disorders. This infection in blood donors potentially indicates a threat of parasite transmission to immunocompromised or immunosuppressed blood transfusion recipients (5–8). Because of in-progress treatments are not efficient and there is no successful vaccine, it is obligatory to perform labors for preventing of *Toxoplasma* transmission to blood recipients for likely incidence of reactivated toxoplasmosis specifically in immunodeficient persons. In this investigation, totally 491 blood samples were collected from blood donors of Razavi Khorasan, Iran. Overall, 200 (40.7%) samples were seropositive for anti-*T. gondii* antibodies; so that 37.5% and 3.2% were positive for IgG and IgM anti-*T.gondii* antibodies, respectively.

Many studies have reported similar *T. gondii* seropositivity in the blood donors of Czech Republic (19), Mexico (22), and southeastern Iran (25, 29, 30); whereas this *T. gondii* seropositivity was more than those reported in India (14), northeast of Thailand (23), and Taiwan (17), and some regions of Iran (26, 28, 30). In contrast, *T. gondii* seropositivity in the blood donors of Razavi Khorasan, Iran was less than the one reported among blood donors in central Iran (26), northeast of Brazil (30), north of India (16), and Egypt (15), where the seroprevalence of *T. gondii* has varied from 50% to 75%. This difference in *T. gondii* seropositivity among the blood donors around the world could be associated to some factors such as geographical and environmental factors, sociocultural habits, transmission routes, sample size in the studied population (9–12). Here, consumption of raw/half-cocked meat is the main risk factor for *T. gondii* seropositivity, indicating that among the blood donors in this study, the consumption of tissue cysts in meat (food-borne transmission) is one of the main routs of infection. Similarly, several studies have reported the food-borne transmission as main infection route in blood donors (26, 34, 35).

By gender, although seroprevalence of anti-*T. gondii* antibodies in female donors were higher male donor, but there is no significant difference in seroprevalence of anti-*T. gondii* antibodies among female and male donors. Recently, seroprevalence of *T. gondii* in female donors was significantly higher than that in males (18, 25). In contrast, *T. gondii* seropositivity was significantly higher among male donors (14, 26). Increasing of anti-*T. gondii* antibodies among female donors might be related to more exposure of females to oocysts and tissue cysts during their daily activities. However, because of a small number of female donors in this investigation, the present findings should be confirmed on a larger sample population.

Similar to the other studies we found that *T. gondii* seropositivity increased with age, indicated age because of increased opportunity for exposure; such a finding was in agreement with those observed in other studies (15, 24, 36).

There was no significant association between education, residence, blood group, raw-milk/egg consumption, and blood transfusion, agriculture activity, and contact with cat and seroprevalence of anti-*T. gondii* antibodies in blood donors.

By Nested-PCR analysis, *T. gondii* DNA was found in all sixteen IgM-positive samples. The presence of parasitemia in IgM-positive healthy blood donors, whereas ensures the likelihood of transmission of *Toxoplasma* through blood transfusion. Parasitemia in two (1.9%) of the IgM-positive subjects were demonstrated from blood donors in southern Iran (27). Moreover, T. gondii DNA was reported in one (9.0%) of IgM-positive samples healthy blood donors from Kerman Province, southeast of Iran (25). These variations could be related to some factors such as the short duration of parasitemia and the low numbers of trophozoites circulating in peripheral blood, which caused false negative results in such cases (37).

## Conclusion

*T. gondii* infection was prevalent among healthy blood donors of Razavi Khorasan Province, in the northeastern of Iran with the overall seroprevalence rate of 40.7% and presence of parasite DNA of *T. gondii* in IgM-positive samples. Ingestion of undercooked meat is associated with increase of seropositivity in the blood donors in the northeast of Iran. The results can be a warning for blood transfusion organizations in order to pay special attention to toxoplasmosis among blood donors and design screening programs as preventive affairs for any probable transmission of toxoplasmosis.

## References

[1] Hill D Dubey J *Toxoplasma gondii*: transmission, diagnosis and prevention. Clin Microbiol Infect. 2002; 8(10): 634–640. 12390281 10.1046/j.1469-0691.2002.00485.x

[2] Robert-Gangneux F Darde ML Epidemiology of and diagnostic strategies for toxoplasmosis. Clin Microbiol Rev. 2012; 25: 264–296. 22491772 10.1128/CMR.05013-11 PMC3346298

[3] Sukthana Y Toxoplasmosis: beyond animals to humans. Trends Parasitol. 2006; 22: 137–142. 16446116 10.1016/j.pt.2006.01.007

[4] Mahmoudvand H Ziaali N Ghazvini H Shojaee S Keshavarz H Esmaeeilpour K Sheibani V *Toxoplasma gondii* infection promotes neuroinflamation through cytokine networks and induced hyperalgesia in BALB/c mice. Inflammation. 2016; 39(1): 405–412. 26490968 10.1007/s10753-015-0262-6

[5] Derouin F Pelloux H Prevention of toxoplasmosis in transplant patients. Clin Microbiol Infect. 2008; 14: 1089–1101. 19018809 10.1111/j.1469-0691.2008.02091.x

[6] Biesiada G Kalinowska-Nowak A Czepiel J Toxoplasmosis—epidemiology, clinical manifestation and infection in pregnant women. Przegl Lek. 2006; 63: 97–9. 16969908

[7] Signorini L Gulletta M Coppini D Fatal disseminated toxoplasmosis during primary HIV infection. Curr HIV Res. 2007; 5: 273–274 17346141 10.2174/157016207780077011

[8] Mahmoudvand H Sheibani B Keshavarz H Shojaee S Esmaeelpour Kh Ziaali N Acetylcholinesterase inhibitor improves learning and memory impairment induced by *Toxoplasma gondii* infection. Iran J Parasitol. 2016; 11 (2): 177–185. 28096851 PMC5236094

[9] Mahmoudvand H Ziaali N Aghaei I Sheibani V Shojaee S Keshavarz H Shabani M The possible association between *Toxoplasma gondii* infection and risk of anxiety and cognitive disorders in BALB/c mice. Pathog Glob Health. 2015;109(8):369–76. 26924347 10.1080/20477724.2015.1117742 PMC4809231

[10] Siegel SE Lunde MN Gelderman AH Transmission of toxoplasmosis by leukocyte transfusion. Blood. 1971; 37: 388–394. 4927414

[11] Shulman IA Parasitic infections and their impact on blood donor selection and testing. Arch Pathol Lab Med. 1994; 118: 366–370. 8166586

[12] Mahmoudvand H Sheibani V Esmaeelpour K *Toxoplasma gondii* infection potentiates cognitive impairments of Alzheimer’s disease in the BALB/c mice. J Parasitol. 2016; 102(6): 629–635. 27513205 10.1645/16-28

[13] Mostafavi SN Jalali Monfared L Toxoplasmosis epidemiology in Iran: a systematic review. J Isfahan Med Sch. 2010; 30(176): 74–87.

[14] Sundar P Mahadevan A Jayshree R *Toxoplasma* seroprevalence in healthy voluntary blood donors from urban Karnataka. Indian J Med Res. 2007; 126(1: 50–56. 17890824

[15] Elsheikha HM Azab MS Abousamra NK Seroprevalence of and risk factors for *Toxoplasma gondii* antibodies among asymptomatic blood donors in Egypt. Parasitol Res. 2009; 104(6): 1471–1476. 19198880 10.1007/s00436-009-1350-z

[16] Yazar S Eser B Yay M Prevalence of anti-*Toxoplasma gondii* antibodies in Turkish blood donors. Ethiop Med J. 2006; 44(3): 257–261. 17447392

[17] Chiang TY Hsieh HH Kuo MC Seroepidemiology of *Toxoplasma gondii* infection among healthy blood donors in Taiwan. PloS One. 2012; 7(10): :e48139. 23133557 10.1371/journal.pone.0048139 PMC3484999

[18] Elhence P Agarwal P Prasad KN Seroprevalence of *Toxoplasma gondii* antibodies in North Indian blood donors: Implications for transfusion transmissible toxoplasmosis. Transfus Apher Sci. 2010; 43(1): 37–40. 20605111 10.1016/j.transci.2010.05.004

[19] Svobodova V Literak I Prevalence of IgM and IgG antibodies to *Toxoplasma gondii* in blood donors in Czech Republic. Eur J Epidemiol. 1998; 14 : 803–805. 9928876 10.1023/a:1007589422080

[20] Makki SM Abdel-Tawab AH Anti-*Toxoplasma gondii* antibodies among volunteer blood donors in eastern Saudi Arabia. J Egypt Soc Parasitol. 2010; 40(2): 401–12. 21246947

[21] Yazar S Eser B Yay M Prevalence of anti-*Toxoplasma gondii* antibodies in Turkish blood donors. Ethiop Med J.2006; 44(3): 257–261. 17447392

[22] Galvan Ramirez ML Covarrubias X Rodriguez R *Toxoplasma gondii* antibodies in Mexican blood donors. Transfusion. 2005; 45: 281–2. 10.1111/j.1537-2995.2004.00442.x 15660841

[23] Pinlaor S Ieamviteevanich K Pinlaor P Seroprevalence of specific total immunoglobulin (Ig), IgG and IgM antibodies to *Toxoplasma gondii* in blood donors from Loei province, Northeast Thailand. Southeast Asian J Trop Med Public Health. 2000; 31: 123–7. 11023078

[24] Alvarado-Esquivel C Mercado-Suarez MF Rodríguez-Briones A Seroepidemiology of infection with *Toxoplasma gondii* in healthy blood donors of Durango, Mexico. BMC Infect Dis. 2007; 7:75–81. 17629901 10.1186/1471-2334-7-75 PMC1940003

[25] Mahmoudvand H Saedi Dezaki E Soleimani S Baneshi MR Kheirandish F Ezatpour B Zia-ali N Seroprevalence and risk factors of *Toxoplasma gondii* infection among healthy blood donors in southeast of Iran. Parasite Immunol. 2015, 37(7), 362–367. 25891186 10.1111/pim.12198

[26] Ormazdi H Sanikhani N Hadighi R Investigation of antibodies (IgG and IgM) against *Toxoplasma gondii* in blood donors referred to Tehran blood transfusion organization by ELISA. Urmia Med J. 2010; 21 (2): 212–216.

[27] Sarkari B Shafiei R Zare M Sohrabpour S Kasraian L Seroprevalence and molecular diagnosis of *Toxoplasma gondii* infection among blood donors in southern Iran. J Infect Dev Ctries. 2014; 8(4):543–7. 24727522 10.3855/jidc.3831

[28] Ferdowsi S Farsi L Tajalli SM Soltani H Seroprevalence Anti-*Toxoplasma gondii* antibodies and anti-Epstein-Barr virus (EBV) antibody among volunteer blood donors referred Gonabad blood transfusion. J Zabol Uni Med Scie Health. 2013; 5(2): 60–9.

[29] Modrek MJ Mousavi M Saravani R *Toxoplasma gondii* seroprevalence among blood donors in Zahedan, southeastern Iran. Int J Infect. 2014; 2(1): e21111.

[30] Zainodini N Zare-Bidaki M Abdollahi S Afrooz M Ziaali N Ebrahimian M Kazemi Arababadi M Molecular and serological detection of acute and latent toxoplasmosis using Real-Time PCR and ELISA techniques in blood donors of Rafsanjan city, Iran. Iran J Parasitol. 2014; 9(3): 336–41. 25678917 PMC4316564

[31] Grigg ME Boothroyd JC Rapid identification of virulent type I strains of the protozoan pathogen *Toxoplasma gondii* by PCR-restriction fragment length polymorphism analysis at the B1 Gene. J Clin Microbiol. 2001;39(1): 398–400. 11136812 10.1128/JCM.39.1.398-400.2001 PMC87743

[32] Chiabchalard R Wiengcharoen JT Sukthana Y Sensitivity and specificity of PCR for the detection of *Toxoplasma gondii* and added to laboratory samples. Southeast Asian J Trop Med Public Health 2005; 36: 408–411. 15916047

[33] Kong QM Lu SH Tong QB Loop-mediated isothermal amplification (LAMP): early detection of *Toxoplasma gondii* infection in mice. Parasit Vectors. 2012; 5:2. 22214421 10.1186/1756-3305-5-2 PMC3280158

[34] Baril L Ancelle T Goulet V Risk factors for *Toxoplasma* infection in pregnancy: a case-control study in France. Scand J Infect Dis. 1999; 31: 305–309. 10482062 10.1080/00365549950163626

[35] Jones JL Dargelas V Roberts J Risk factors for *Toxoplasma gondii* infection in the United States. Clin Infect Dis. 2009; 49: 878–84. 19663709 10.1086/605433

[36] Coelho RA Kobayashi M Carvalho LB jr Prevalence of IgG antibodies specific to *Toxoplasma gondii* among blood donors in Recife, Northeast Brazil. Rev Inst Med Trop Sao Paulo. 2003; 45: 229–231. 14502353 10.1590/s0036-46652003000400011

[37] Nimri L Pelloux H Elkhatib L Detection of *Toxoplasma gondii* DNA and specific antibodies in high-risk pregnant women. Am J Trop Med Hyg. 2004;71(6):831–5. 15642979

